# Should recipient parents have access to gamete donors’ raw genomic data? Clinical, legal, and ethical considerations

**DOI:** 10.1007/s10815-025-03666-4

**Published:** 2025-09-27

**Authors:** Shiri Shkedi-Rafid, Aviad Raz, Maya Sabatello, Barbara Prainsack, Roy Gilbar

**Affiliations:** 1 Department of Genetics, Hadassah Medical Center, The Hebrew University of Jerusalem, Jerusalem, Israel; 2 Department of Sociology and Anthropology, Ben Gurion University of the Negev, Beer Sheva, Israel; 3 Department of Medicine, Center for Precision Medicine and Genomics, Columbia University Irving Medical Center, New York, NY USA; 4 Department of Medical Humanities and Ethics, Division of Ethics, Columbia University Irving Medical Center, New York, USA; 5 Department of Political Science, University of Vienna, Vienna, Austria; 6 Law School, Netanya Academic College, Netanya, Israel

**Keywords:** Game donation, Genetic testing, Raw genomic data, Informed consent, Re-contact, Privacy

## Abstract

**Background:**

Genomic sequencing yields vast amounts of data, and the access of patients and research participants to their raw genomic data raises ethical and practical dilemmas.

**Purpose:**

This paper aims to examine a challenging, underexplored question: whether gamete donors' raw data should be provided to recipient parents.

**Methods:**

Using a clinical case, we explore the key ethical, legal, and clinical implications of such access, weighing the advantages, disadvantages, and potential alternatives.

**Results:**

Ethical implications include the feasibility of meaningful informed consent from donors for complex genetic testing, sometimes years after donation; privacy considerations; the type of medical information recipients can or should hold on donors; potential conflicts of interest between the donor and the child; and the potential for raised costs and commercial interests. Clinical implications involve the implementation of systems of storing donors' raw data and devices of re-contacting past donors. Legal aspects include  the informed consent of gametes donors to disclose their raw data to recipient parents and privacy protections, including their right to keep their raw data in confidence.

**Conclusion:**

We advocate a cautious approach that favors clinically mediated access over unrestricted parental access.

## Introduction

Genomic testing, mainly exome and genome sequencing, is becoming an integral part of clinical care in all settings: from prenatal diagnosis to newborn screening, pediatric testing, and testing adults. Exome sequencing focuses on the coding regions of the genes (exons), where most known disease-causing mutations are found. Genome sequencing examines the entire DNA sequence, including both coding and non-coding regions, and can therefore uncover rare variants that may be missed by exome sequencing. Testing is mainly carried out to provide a diagnosis in fetuses/children/adults with clinical indications suggestive of genetic etiology. However, the uptake of genome testing in healthy fetuses and adults is also growing [1, 2]. The most common type of genomic testing today is exome sequencing. Exome sequencing can be performed on the proband only (singleton) or together with both biological parents (trio). Although the diagnostic yield of a trio exome is only slightly higher than that of a singleton [3, 4], comparing the proband’s exome to their biological parents can assist in classifying uncertain genetic variants identified in the proband.

Genomic sequencing yields enormous amounts of data. To date, conceptual and empirical research have focused mainly on the types of variants that should be disclosed from such tests, mainly with regard to uncertain, probabilistic, and secondary findings (i.e., highly penetrant, pathogenic genetic findings unrelated to the clinical indication for testing, yet important to the health of the tested individual/their offspring). The American College of Medical Genetics and Genomics (ACMG) recommends the disclosure of pathogenic variants in a set list of genes (currently 84) associated with preventable/treatable conditions [5].

Another aspect of genomic testing, much less studied, is the return of raw genomic data to patients undergoing genetic testing and to participants in genetic studies. Raw genomic data refers to the sequence of DNA letters prior to any interpretation. While the classification of a variant may vary between laboratories and over time, the raw genomic data itself remains the same. Periodic re-evaluation and analysis of the raw data can increase the diagnostic yield in individuals without an initial molecular diagnosis, as novel disease-causing genes continue to be identified [6], and analysis tools are regularly upgraded and improved.

Fairness, reciprocity, and respect for autonomy have been highlighted as important ethical reasons for allowing research participants and patients to access their raw genomic data [7]. Precluding patients from accessing their own raw data has been described as paternalistic [8]. Empirical studies that explored the views of (mainly European, healthy) publics show that many indicate a hypothetical interest in receiving their genomic raw data if offered to them. These participants provided two main reasons for this interest: First, they consider this data their own, and second, they may want to interpret the data using online sources or other experts [9]. Yet, these studies did not explore “hard cases,” such as access to fetal genomic data, or third-party gamete donors.

Donor-assisted conception is used by parents who experience infertility, same-sex couples, or single parents. According to the American Society for Reproductive Medicine and the European IVF-monitoring Consortium, the use of gamete donation has increased over the past several decades [10, 11]. With the increasing prevalence of diverse families that are created through gamete donation and a constant rise in age-related infertility [12], the number of third-party pregnancies utilizing gamete donation is only likely to increase.

Ethical considerations regarding genetic testing of donors (both sperm and oocyte), developed and published by the European Society of Human Reproduction and Embryology (ESHRE) in 2014 [13], principally focused on how expanded donor genetic testing should be. It concluded that testing should mainly include testing for autosomal recessive and X-linked conditions (in oocyte donors), which have no relevance to donors’ own health but solely to that of their offspring. Of note, while the results from carrier screening are available to fertility clinics or sperm banks, donors, and recipients, donors’ raw genomic data are not available to any of them. The raw data are either stored at the genetics lab or destroyed after completing the carrier screening test. Advanced genomic tests may be recommended later on, in offspring conceived with the assistance of gamete donation, whereby donors’ genomic data may assist in providing a diagnosis in the fetus or child. This gap between the scope of pre- and post-donation genetic testing means that donors’ DNA or raw genomic data should either be securely stored for potential future analysis or donors should be re-contacted if additional genetic testing becomes warranted.

In 2022, ESHRE published recommendations for all parties involved in reproduction with donated gametes (both sperm and oocyte) on information provision, with an emphasis on direct-to-consumer genetic testing done on donor-conceived offspring to ascertain the donors’ identity and find genetic siblings. They recommend that potential donors should be informed of current and possible future legislation and technology (such as ancestry testing via direct-to-consumer companies) that could increase the risk of re-identification [11]. Recommendations also relate to donors’ responsibility to update fertility clinics/gamete banks of new medical information about themselves/their families and possible future contact with donors for additional medical information. Although guidelines primarily address the responsibilities of donors, they also recommend informing donors that they may be contacted by the fertility center or gamete bank for additional medical information, such as reports of unexpected diseases in offspring.

Significantly, many of the principles raised both in 2014 and in 2022 apply to the issue of access to the raw genomic data of gamete donors. These include the feasibility of meaningful informed consent for complex genetic testing, exposing donors to societal discrimination and/or stigmatization, and expanded genetic information on donors as a potential cause for raised costs and commercial interests. Nevertheless, there is neither a mention nor a specific discussion regarding the recipient parents’ access to donors’ raw genomic data. Unlike natural conception, where both parents are commonly available for additional genetic testing, recipients of gamete donation may desire having access to the donor’s raw genomic data to enable future analysis should it be needed (e.g., in situations in which abnormal findings are identified during pregnancy or in childhood).

Professional guidelines currently address the types of genetic tests performed on potential donors and the results from these tests that are disclosed to recipients. Concrete guidelines regarding the sharing of raw genomic data in clinical settings and in various life stages (including embryos) are currently missing, including in the context of gamete donation. Consequently, each genetic lab and sequencing project determines its own practice [9, 12, 14]. This paper seeks to fill this gap by laying out how key challenges could be addressed and by identifying relevant considerations in this process.

Altogether, we advocate a cautious approach that favors clinically mediated access over unrestricted parental access.

Although practices and guidelines may differ between sperm and oocyte donation, this paper addresses considerations that are largely shared across both types of gamete donation.

## The case of Anna and George: consenting to the sharing of raw data

The following case, based on an actual clinical request, illustrates a potential situation unique to genomic testing in gamete donors:

*Anna and George are in the process of anonymous oocyte donation *via* an agency that coordinates the donation process. The donors’ screening process includes whole-exome sequencing to identify carriers for X-linked and autosomal recessive conditions. Anna and George receive the anonymous exome report, with the donor’s consent. They are also interested in receiving the raw data from the donor’s exome sequencing and approach the agency with this request. They explain that this way, they will not be dependent on re-contacting the donor for additional testing if concerning findings are identified in the fetus or in the born child.*

This scenario raises questions that clinicians, gamete donors, and recipient parents may increasingly face: Should the raw data of gamete donors be provided to recipient parents, meaning that the latter may hold medical information about the donor before the donor themselves are aware of it? What are the advantages and disadvantages of doing so? And what alternatives exist?

As the discussion in this article shows, while the application of leading bioethical and legal principles (mainly autonomy, beneficence, confidentiality, and justice) raises dilemmas that are similar in other areas, the specific characteristics of genomic testing in gamete donors make these dilemmas unique. In the following section, we will use the key bioethical principles to examine this scenario.

### Autonomy

Autonomy is considered a highly influential principle when discussing communication and access to genetic information. It reflects several aspects of one’s interests in controlling life and health. These include the interests in making informed and independent decisions (legally known as making informed consent decisions) and the interest in controlling access to one’s personal information (known as privacy and confidentiality). In this part, we will deal with these aspects.

From the perspective of voluntariness and informed consent, it could be argued that if a donor receives thorough counseling about the various aspects of access to their raw data by the recipient parent(s) and provides their informed consent for such data sharing, then this practice is acceptable [8, 15]. Following this rationale, if the donor agreed, Anna and George would be able to receive the donor’s raw data. However, in our context, the following two main aspects of consent arise.

#### Can consent address future scenarios?

Consent is commonly used in medical practice as a binary option (yes/no) with a limited number of possible outcomes (e.g., undergoing a medical procedure, receiving a treatment) that the person giving consent is supposed to assess in the present (i.e., at the moment when they give consent) [16]. In the context of gamete donation, reinterpretation of the donor’s raw genomic data could occur years after donation. In recent decades, genetic testing has evolved rapidly, and assuming that it will continue to do so, it may generate future unforeseen scenarios that the person giving consent for their data to be shared could not have possibly envisaged.

Reanalysis over a long period of time is particularly relevant to genomic research, given the aforementioned increase in diagnostic yield via reanalysis. Existing guidelines, however, are still lacking in their specification regarding the process of reanalysis and how re-contact should be operationalized [17]. To date, although there are professional ethical guidelines that address this issue, there is no general legal duty to re-contact patients who underwent genetic testing for their own purpose or to those who volunteered to participate in research. This is, for example, the situation in the UK [18], Canada [19], and the USA [20]. Moreover, whereas genomic research is aimed at achieving a highly desired diagnosis in an individual with demonstrated symptoms, gamete donors are generally healthy, and reanalysis of their genomic data is mainly done to assist in diagnosing an offspring whom they may not know.

Would donors’ consent at the time of donation indeed reflect their view later in their lives? Two central dilemmas, which have received substantial scholarly attention in other contexts, are as follows: (1) What should be the case if the donor changes their view from the time of signing the consent form (e.g., regarding reinterpretation of the raw data)? (2) What should be the decision in a situation that is not described in the consent of the donor? These questions may be critical if a serious and actionable problem is discovered.

Empirical data on these aspects is limited. Most relevant for our discussion, Daniels et al. [21] show that nearly half of sperm donors included in their study (*n* = 32) changed their views regarding their willingness to be identified to offspring in the future compared to attitudes recorded at the time of donation. A change in donors’ views was seen in both directions—a willingness to be more open or anonymous.

Legally, at least in some jurisdictions, consent which addresses future scenarios is considered binding if no new evidence indicates that they changed their mind [22]. It could be argued that, legally, it is possible to require clinicians who seek raw data sharing consent from donors to be transparent about the impossibility of foreseeing all future uses and of emphasizing that donors would not be able to change their minds once the raw data was shared with the recipients.

The same problem exists in other areas in health and medicine (including in genomic research), and it is not considered an impediment for clinical practice: There are situations in which specific consent is given at a particular time, even though the range of future scenarios is unknown. Active withdrawal is the only way to subsequently revoke the initial consent. For example, men who agree that their female partner uses their sperm to have a child after their death (known as posthumous use of sperm) know that their partner would be able to use it as long as there is no new evidence to the contrary [23–25]. Similarly, people who sign a consent form stating that ten years after their last IVF treatment, their surplus embryos would be donated to research are bound by their consent as long as they do not approach the lab within this period to withdraw their consent [26]. Other examples concern advance directives in the context of end-of-life decision making.

Consequently, genetic testing in gamete donors could facilitate a choice of a consent model that can address the aforementioned challenges. In particular, the following several consent models have been discussed.

The first model is *specific consent* to each individual test [27, 28]. This requires that donors be re-contacted if new tests/uses of their data are being considered, sometimes years after donation. This would allow gamete donors, like the donor in our hypothetical scenario, to make decisions at a time when the information is relevant to them or their offspring’s health. The disadvantage is that it may be difficult or unfeasible to contact the donor, e.g., if they have changed contact information, died, or otherwise are lost for follow-up. Additionally, this is time-consuming for fertility clinics and may not be financially viable.

Another potential model is *dynamic consent* [29]. This model offers a personalized, online consent and communication platform which allows bi-directional, dynamic, and continuous communication between clinicians and donors. The disadvantages include the (often complex) technical requirements, as well as the digital capabilities required on the side of the donors. There may also be the additional challenges of explaining new updates on genetic testing via an app (which have not yet been tested for efficacy and retention of information), as well as the costs associated with both the technical and educational challenges.

Finally, the* meta-consent* [30–32] model could be offered as well, being an approach which enables individuals to express preferences regarding which type of consent they want to give for which type of test (for example, broad consent to tests that are only relevant to offspring’s health, specific consent to tests that may affect their own health).

Regardless of the type of consent, consent to genetic testing and its implications is part of a long process that people undergo to become donors. This brings us to the second aspect of consent.

#### Can donors be adequately informed?

Clinicians must ensure that gamete donors are adequately informed about current (at the time of donation) and future aspects of genetic testing. Studies exploring the perspectives of participants in genomic research have highlighted challenges associated with determining how much information to provide to participants during the informed consent process. Whereas some participants valued a lengthy explanation, others felt that the time and amount of information provided to them were overwhelming [33]. Regardless, studies suggest that retention of information included in consent forms for research participation is limited [34–36]. The question of what constitutes “sufficient” information, especially in the context of genomic tests in gamete donors, is not yet answered.

Outram et al. [17] explored how reanalysis of genomic raw data is presented to parents enrolled in a prenatal and pediatric genomic study in California, USA. These parents were offered free genomic sequencing because their child or fetus presented with an undiagnosed deleterious condition that was suspected to be genetic in origin. Based on interviews conducted a few months after recruitment, they showed that study participants were unclear about the likelihood that reanalysis will be conducted, the process of initiating reanalysis, and whether they would receive revised results. Niemiec and Howard [37] examined whether gamete donors taking part in genomic editing studies are genuinely informed about the implications of genomic tests done on their gametes. They found that understanding and engagement required a combination of both verbal communication and detailed consent forms. They also encouraged involving a genetic health professional in discussions about genetic findings.

However, this phenomenon, which is not unique to gamete donation, does not necessarily undermine how adequately informed the donor’s consent is. For example, genetic counseling usually involves relatively long encounters between the clinician and the patient. In this encounter, a large amount of information is communicated to the patient. Oftentimes, the information is complicated and difficult for people to understand and process. Yet, the patient’s consent to undergo testing after this meeting with the clinician is considered legally valid [38, 39]. It could therefore be helpful to include a clinical geneticist/genetic counsellor in the process of obtaining informed consent from the donor. Furthermore, fertility clinics may consider dividing the consent process into a few meetings to allow a more thoughtful process. If significant time and efforts are not invested in counseling donors, truly informed consent cannot be assumed.

Another important aspect in the context of gamete donation is making sure that donors do not experience pressure to consent to sharing their raw genomic data. Empirical studies on donors’ attitudes toward expanded carrier screening (ECS) show that while some perceive the free access to data that could inform their own reproduction as beneficial, others may find it overwhelming and anxiety-provoking. Other donors may even decline donation because they do not wish to undergo ECS [40, 41]. Mukherjee [42] found that oocyte donors felt pressured to make the decision and insufficiently informed, indicating that clinicians did not genuinely address their concerns, and that the experience resulted in a sense of increased medical objectification of their bodies. Pressure to consent to sharing their raw genomic data may be particularly significant in donors whose main motive for donation is financial need, such as egg donors who receive partial or full coverage of IVF cycles in return for donation [40, 43]. Cohen et al. [44] showed that donors would agree that their donation would be identifiable to the offspring in the future if more money would be offered. Similarly, donors who consent to sharing their raw data may be more expensive, and the additional burden on the donors would be financially compensated. This may increase the exploitation of donors, the commodification of gametes, and disparities in access, which have already been highlighted as ethical concerns regarding gamete donation [45].

The discussion about consent to sharing raw genomic data with others may also raise another important ethical aspect of autonomy, namely, the donor’s privacy.

#### Violating the donor’s privacy

It has been shown that individuals could be re-identified from de-identified sequence datasets [46–48]. The three main settings in which genomic data are constructed and stored are healthcare entities, research projects, and direct-to-consumer companies. In the case of an undiagnosed disease or in search of treatment, the benefit of genomic testing (via healthcare or research entities) may outweigh the risk of privacy loss.

From a legal perspective, the privacy of one’s genomic data receives substantial protection (i.e., GDPR) [49]. However, in some Western countries, the disclosure of identifiable genetic and genomic data is justified when it benefits the health of blood relatives [50]. Thus, the question here is whether and how the donor’s data can benefit the child and parents and whether such benefits outweigh the potential harms to the donor. The latter harms may emerge from the use of the donors’ genomic data to examine ancestry [51] or for commercial purposes [52] in the absence of concrete consent for such specific genomic analyses.

Moreover, from both bioethical and legal perspectives, the mere risk of re-identification of their identity may compromise their fundamental rights to privacy and anonymity when they were uninformed about the full risk or could not reasonably understand it, given the open nature of future sequencing technologies. These infringements can be considered harms that clinicians should try to avoid [15]. However, it could be argued that potential breach of donors’ privacy is not convincing if donors have undergone a consent process that explains this risk or when it is weighted against the potential benefits to the health of the resulting child. Nevertheless, we argue that privacy loss when the data is used for non-medical reasons (e.g., using PRS to assess educational attainment, as detailed in the next section) cannot be justified.

Moreover, when the identity of the donor is, or becomes, known to offspring/recipients (in cases of known donors—donors who donate to family members or friends) or will be known in the future (in countries where donor-conceived people are legally allowed to find out the identity of the sperm donor when they reach the age of 18) [53], access to donors’ raw genomic data could violate donors’ privacy since sensitive and private information could be accessible to recipients/offspring.

### Nonmaleficence and beneficence

Clinicians are obliged to do their utmost not to harm their patients and to improve their overall condition [15]. In gamete donation, we view both the donor and the conceived child (once born) as patients, which makes it difficult to balance the child’s best interests against those of the gamete donor. In addition, bioethicists stress that harm has several dimensions (e.g., physical, emotional, infringement of rights) [15, 54].

The overall goal of genetic testing in gamete donors is to prevent transmission of genetic disorders to donors’ offspring. According to current guidelines, donors are mainly tested for genes associated with severe, childhood-onset, autosomal recessive and X-linked conditions (in egg donors). Testing donors for autosomal-dominant conditions, especially those with incomplete penetrance and late onset, is not currently recommended, unless the donors’ family history suggests the need for additional genetic testing for the donor [10].

Once recipient parents, like George and Anna in our case, receive the donor’s raw genomic data, they may extract information on the donor that does not adhere to current practice and clinical guidelines (i.e., pathogenic variants in genes associated with autosomal-dominant conditions, not included in the preliminary analysis and report). Such information could range from medically valuable information (for instance, a pathogenic variant in the *BRCA1* or *BRCA2* genes, conferring a high risk for breast and/or ovarian cancer for the donor) to findings with limited clinical utility, such as polygenic risk scores (PRS) for multifactorial conditions. PRS is an estimation of a person’s genetic risk for a certain condition, based on the combined effect of many common genetic variants. Each of these variants contributes a small amount to the overall risk, and when added together, they help predict the likelihood of developing complex diseases like diabetes, heart disease, or cancer. PRS is not diagnostic but can indicate higher or lower genetic susceptibility compared to the general population. PRS can also be used to predict non-actionable conditions, such as risks for Alzheimer’s disease. The use of PRS for medically actionable conditions in specific populations is already done in national studies (such as the eMERGE study in the USA) [55]. Nevertheless, PRS in the setting of pre-implantation genetic testing (PGT) [56] (i.e., genetic testing carried out on embryos obtained via in vitro fertilization (IVF), before they are implanted in the uterus of embryos free from the tested disease) is currently disapproved by professional bodies such as the European Society of Human Genetics (ESHG) [57] and (ACMG) [58, 59]. That said, in the USA, the legal and ethical tradition of reproductive decisions emphasizes parental choice [60], enabling private companies to provide services that do not meet legal and ethical criteria. Other countries, such as South Africa, Singapore, and China, are less restrictive about (PGT) using PRS [61–63]. Individuals seeking to perform PGT for multifactorial conditions may extract PRS data from donors’ raw data via private (mainly direct-to-consumer) companies, not mediated by clinicians [64]. The validity of such knowledge is questionable [65] and may also jeopardize donors’ privacy as detailed in the following section. Therefore, while clinical advantage is likely to be limited (to either the donor or the child), donors’ initial consent to the type of data obtained from their genetic testing (i.e., definite, actionable) could be violated.

Information about late-onset conditions, such as increased cancer risk, is generally not tested for in fetuses or children. Professional guidelines emphasize respecting the future autonomy of the child to decide, once mature enough, whether and when to undergo such testing [66, 67]. When recipient parents obtain this information early, it may compromise the donor-conceived individual’s right to an open future.

## Summary and recommendations

Although the scenario and implications discussed here may be relatively rare at present (though we witnessed such requests in clinical settings in Israel), we believe that such questions will increase with the ever-expanding use of both genetic testing and gamete donation. Albeit its size, Israel has been a key user of assisted reproductive technologies, including gamete donation [45]. Therefore, the dilemmas arising in Israel may be relevant in other countries.

Beyond the financial element for both donors and the fertility clinic, gamete donation is also an altruistic act. Once donors agree to provide their gametes, they should be aware that their medical information resulting from genetic tests might be used for health purposes related to the conceived child—and agree to this in the consent process. The potential benefits of sharing donors’ genomic data with recipients include the ability to identify medically valuable information relevant to both the donor’s and offspring’s health, facilitating rapid reanalysis if needed for clinical diagnosis in the offspring, and reducing costs for fertility clinics or sperm banks by avoiding the need to store genomic data or re-contact donors. However, these benefits must be weighed against possible harms, including violations of donors’ privacy, overriding donors’ original consent for additional genetic testing, overlooking their interests in favor of those of recipients—particularly when recipients are the ones funding the procedure—and the risk of identifying findings with limited or uncertain clinical utility (Table 1).

**Table 1:** Potential benefits and harms of sharing donors’ genomic data with recipient parents

Potential benefits	Potential harms
Enables identification of clinically relevant information for donors’ and offspring’s health	Possible violation of donors’ privacy
Facilitates rapid reanalysis of donors’ genomic data when needed for clinical diagnosis in offspring	Consent given at the time of donation may not reflect the donor’s future views and cannot anticipate unforeseen uses of genomic data arising from advances in testing and reanalysis
Reduces costs for fertility clinics/sperm banks by eliminating the need to re-contact donors or repeat testing	Risk of identifying findings with limited or uncertain clinical utility

Even if recipients receive access to donors’ raw genetic data, additional complexities arise, such as their responsibility toward donors, in case they have important medical information on them. Should they inform the fertility clinic of results obtained from analyzing the raw data, and if so, what circumstances should give rise to such a responsibility? Should the donor have a right to genetic counseling, and by whom? Who should pay for it? What if the donor claims to be harmed by the information? These are complicated questions that also involve practical challenges which need to be addressed.

Given the complex dilemmas that may arise from genetic information and tests done in donors and children born with the assistance of gamete donation, clinics should serve as a good-faith broker in cases of medical need of the child; this will eliminate the need for the parents to have access to the data and balance out the privacy risks and medical benefits.

A possible solution may be to retain donors’ genomic data at genetic labs, with a strict access policy (clinicians only), so that reanalysis can take place in particular cases of a child’s clinical needs and only be used within a very narrow scope. The possibility of re-contacting donors for medically valuable information would still be relevant, and the possibility of re-contacting donors should be discussed in the informed consent process at the time of donation to assure that future contact is not perceived as violating the donors’ privacy. Re-contacting donors should be done by the fertility clinic that facilitated the donation based on clinical judgement (including that of a geneticist/genetic counsellor) and not on the recipients’ request. Fertility clinics should develop mechanisms for keeping up-to-date contact information on donors. This solution is not without weaknesses, especially in countries such as the USA, where gamete donation is privatized: There is no national registry of gamete donors, and donors can donate in multiple clinics, hence potentially requiring each clinic to separately re-contact a donor (and each of them may also have a different incentive and threshold for when such re-contact would be justified). These considerations may explain the lack of a legal duty to do so.

Ultimately, however, addressing the issue is important to protect the well-being—even if only in the form of information—of patients, both donors and future children. Therefore, it requires the concerted effort of professional societies to take a stand on the issue and draw clear guidelines alongside policymakers who can require that sufficient resources are allocated to address the financial consideration as a reason to preclude re-contact and reanalysis.

Finally, more empirical research is needed on donors’ understanding of the implications of sharing their raw genomic data with recipients, re-contacting donors, and recipients’ perceptions of the utility of obtaining the donors’ raw data.
